# Prospective Assessment of Transforaminal Lumbar Interbody Fusion (TLIF) in Spondylolisthesis: A Radiological and Functional Outcome Study in an Indian Population

**DOI:** 10.7759/cureus.67880

**Published:** 2024-08-26

**Authors:** Sai Preeth, Vijayanand B, Rishab C, A Robin, Sidharthan Dhasarathy

**Affiliations:** 1 Department of Orthopedics, Sri Ramaswamy Memorial (SRM) Medical College Hospital and Research Centre, Kattankulathur, IND; 2 Department of Orthopedic Surgery, Sri Ramaswamy Memorial (SRM) Medical College Hospital and Research Centre, Kattankulathur, IND; 3 Department of Orthopedics and Traumatology, Sri Ramaswamy Memorial (SRM) Medical College Hospital and Research Centre, Kattankulathur, IND

**Keywords:** oswestry disability index, functional outcomes, radiological outcomes, transforaminal lumbar interbody fusion, spondylolisthesis

## Abstract

Introduction: Spondylolisthesis is a common spinal condition in which one vertebra slips over another, leading to pain and disability. Transforaminal lumbar interbody fusion (TLIF) has emerged as a surgical option for addressing spondylolisthesis; however, limited research exists, especially in the Indian context, evaluating its radiological and functional outcomes.

Objective: The study aimed to evaluate the radiological and functional outcomes of TLIF in spondylolisthesis using standardized scoring systems, to evaluate the sagittal balance of the spine radiologically in patients who have undergone TLIF for spondylolisthesis, and to evaluate the correlation between the functional and radiological outcomes after TLIF.

Methods: This prospective observational study included spondylolisthesis patients undergoing TLIF at SRM Medical College Hospital and Research Centre from August 2022 to August 2024. Criteria included Meyerding grade 1-4 spondylolisthesis, single-segment fusion, and willingness for 12-month follow-up.

Results: Forty-five patients were included with age 36.6 ± 12.2 years, with 73.3% being female. L4-L5 is the most common level affected in 21 patients (46.7%). Significant improvements were observed in pelvic tilt 19.07 ± 2.05, sacral slope 30.6 ± 4.4, segmental lordosis 18.4 ± 1.4, lumbar lordosis 57.1 ± 1.8, sagittal vertical axis (SVA) 2.5 ± 0.3, Visual Analog Scale for pain 0.4 ± 0.5, and Oswestry Disability Index (ODI) scores 5.23 ± 2.6 postoperatively (p < 0.05). At one-year follow-up, 84.4% of patients had good-to-excellent outcomes, and 44.4% had definitive fusion according to modified Lee criteria. However, there was no correlation between ODI score and grade of listhesis, pelvic incidence (PI), or SVA of the spine (p > 0.05).

Conclusion: This study provides valuable insights into the effectiveness of TLIF surgery in addressing spondylolisthesis, both in terms of radiological and functional outcomes. However, there was no correlation between improvement in functional and radiological parameters (PI vs. ODI, SVA vs. ODI). TLIF appears to offer significant improvements in patient well-being and quality of life. These findings contribute to understanding TLIF's suitability as a treatment for spondylolisthesis and can inform clinical practice, ultimately benefiting patients suffering from this condition.

## Introduction

Spondylolisthesis is when one vertebra slips over another, often occurring in the lumbar region of the spine. This condition, though insidious and painful, significantly impacts those it afflicts. It manifests in various forms, with degenerative and isthmic spondylolisthesis being the most common. Degenerative spondylolisthesis results from age-related spinal degeneration, displacing vertebrae, while isthmic spondylolisthesis usually stems from stress fractures of pars interarticularis, often in childhood or adolescence. Both forms cause severe lower back pain, radiating from the lower back to the legs, and disrupt spinal stability, hindering daily activities [1,2].

The prevalence of spondylolisthesis is a matter of concern, affecting 6%-7% of the population, with grade I spondylolisthesis making up 75% of cases [3]. It typically occurs at the L5-S1 level, causing the L5 vertebral body to move forward over the S1 vertebral body. Many individuals experience a significant reduction in their well-being due to the associated pain and discomfort, which can lead to disability and affect work, social life, and self-care. For those living with spondylolisthesis, tasks like walking, standing, and bending become arduous, impacting employment and social interactions, and disrupting sleep. Effective treatment is imperative, as living with spondylolisthesis is not a viable option [3].

Currently, posterior pedicular screw fixation combined with interbody fusion is the preferred treatment for spondylolisthesis. Various interbody fusion methods exist, including anterior lumbar interbody fusion, posterior lumbar interbody fusion, lateral lumbar interbody fusion, oblique lumbar interbody fusion, and transforaminal lumbar interbody fusion (TLIF). Minimally invasive TLIF is superior to open TLIF but has advantages and disadvantages [4-7]. Open TLIF is widely performed due to its minimal impact on nerve roots and the spinal cord's protective covering (dura), lower risk of postoperative nerve inflammation (radiculitis), instrument availability, and familiarity of the surgeon [8-10].

Although several studies have examined TLIF's effects on spinopelvic parameters and correction levels in different ethnic groups, there is limited data specific to the Indian population [11-13]. Thus, this study seeks to evaluate TLIF's efficacy in addressing spondylolisthesis challenges by assessing its radiological and functional outcomes. The study fills gaps in understanding and informs healthcare professionals, policymakers, and the healthcare community about TLIF's suitability as a treatment for spondylolisthesis, ultimately enhancing patients' quality of life. The study aims to evaluate the functional outcomes of patients with spondylolisthesis who have undergone TLIF using standardized objective scoring systems and to investigate radiological outcomes in these patients.

## Materials and methods

This prospective observational study investigates the impact of TLIF on the radiological and functional outcomes in patients with spondylolisthesis, assessing its efficacy in improving spinal stability and functional well-being. The research spans from August 2022 to August 2024, allowing for a comprehensive assessment of radiological and functional outcomes in patients with spondylolisthesis following TLIF treatment. Patients diagnosed with spondylolisthesis who have undergone TLIF within the Department of Orthopedics at SRM Medical College Hospital and Research Centre were included. Consecutive patients with degenerative/isthmic spondylolisthesis planned for TLIF were selected based on inclusion and exclusion criteria, with informed written consent obtained from all participants.

Inclusion criteria included age over 18, Meyerding grade 1-4, degenerative or isthmic spondylolisthesis diagnosis, single-segment fusion, radicular symptoms, back pain consistent with radiologic findings, and unsuccessful conservative therapy for at least six weeks. Exclusion criteria included pregnant women, multilevel TLIF, history of previous spine surgery, presence of degenerative scoliosis, preoperative frontal imbalance, and other neurological diseases such as post-traumatic paraplegia or infection.

The sample size of 45 was calculated based on a similar study by Ould-Slimane et al., considering the expected mean and standard deviation of preoperative and postoperative pelvic incidence (PI) [11]. The level of significance and power were taken as 5% and 80%, respectively. Around 45 patients diagnosed with spondylolisthesis attending our hospital were randomly selected using a computer-generated randomization schedule to ensure unbiased selection. This process involved assigning numbers to eligible patients, and a random number generator was used to select participants for the study using the SPSS version 2.0 software (IBM Corp., Armonk, NY). This method minimized selection bias and ensured that the sample represented a broad spectrum of spondylolisthesis cases. Patients were followed up in three months, six months, and one year. Data included patient demographics, preoperative radiological parameters, surgical procedures, and clinical assessments.

Preoperative radiological evaluation

Preoperative radiological assessments included standing full-length spine radiographs, MRI of the lumbar spine with whole spine screening, and various measured parameters: PI, pelvic tilt (PT), sacral slope (SS), segmental lordosis (SL), lumbar lordosis (LL), and sagittal vertical axis (SVA) [13-15].

Surgical procedure

The open TLIF surgical procedure was performed by a spine surgeon under general anesthesia, with preoperative antibiotics administered. Patients were positioned in the prone position on a Jackson table, and the surgical site was prepared and draped following standard sterile procedures; the proper spinal level was identified using a C-arm. Self-retaining retractors were used to ensure visibility and accessibility. Bilateral pedicle screws were inserted for stabilization, and the TLIF procedure commenced on the symptomatic side. The inferior facet of the cephalad (top) vertebra was extracted using a 10-mm osteotome. The interlaminar gap was then diverted by using a laminar spreader, and the superior facet of the caudal vertebra was removed for subarticular decompression. Cancellous autograft was harvested, and a box-shaped incision was made on annulus fibrosis to gain access to the disc space. The cage with the harvested graft was inserted into the disc space, and decompression was verified using imaging.

Postoperative assessments

**Figure 1:**
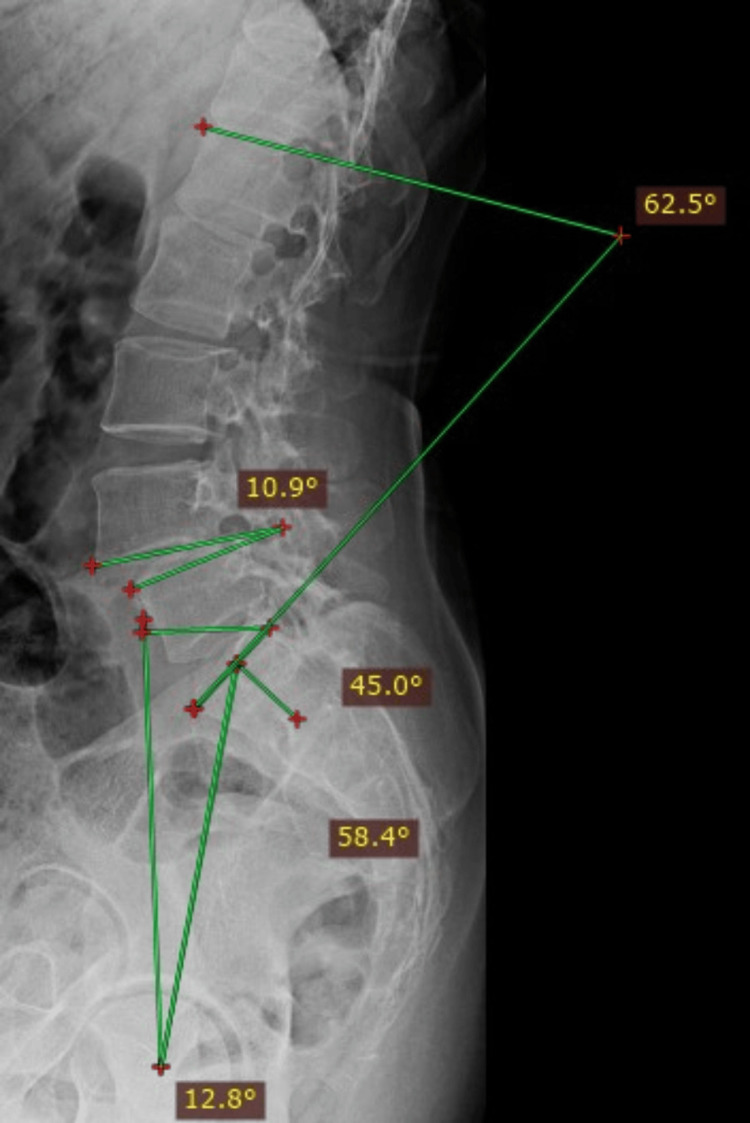
Measurement of spinopelvic parameters in a preoperative spondylolisthesis patient

**Figure 2:**
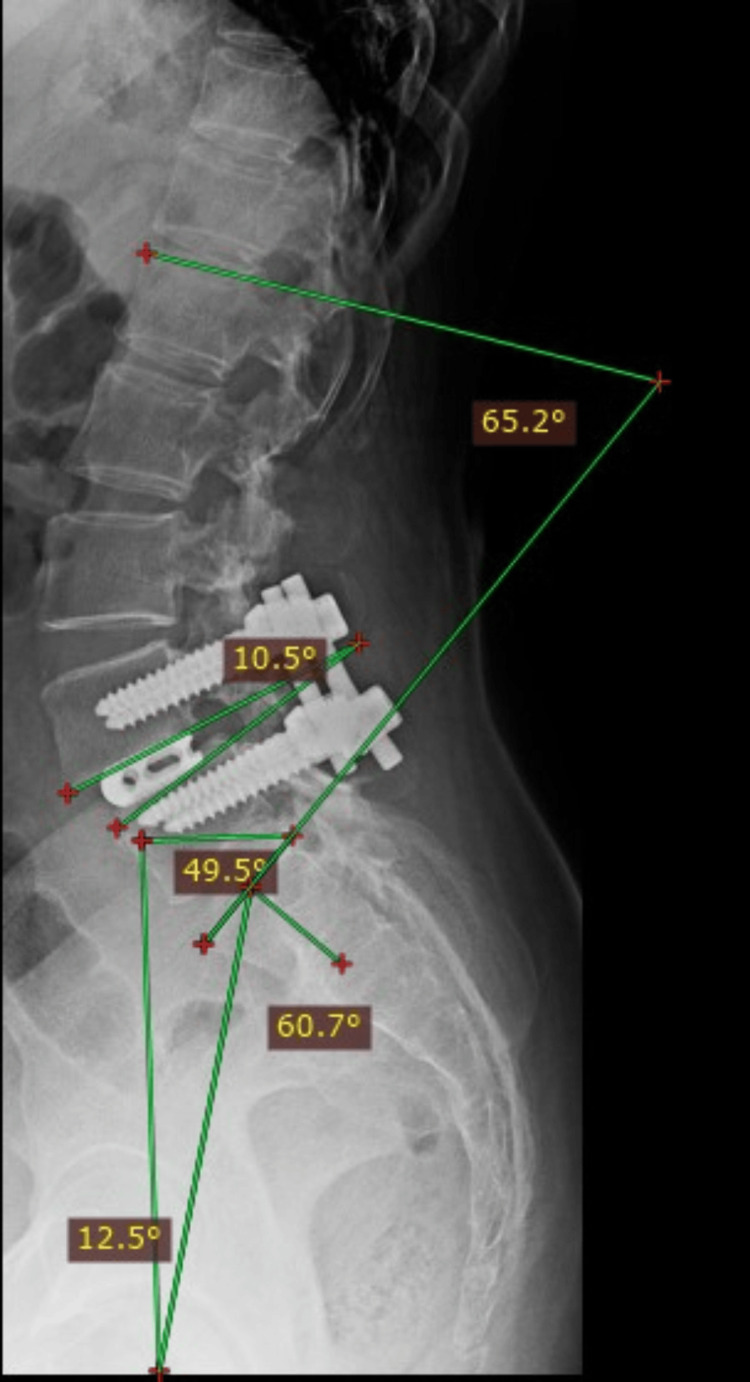
Measurement of spinopelvic parameters in the last follow-up following TLIF surgery TLIF: transforaminal lumbar interbody fusion

Postoperative assessments included measuring pain levels using the Visual Analog Scale (VAS) and functional well-being using the Oswestry Disability Index (ODI) [16]. Patients were also assessed using the Macnab criteria and modified Lee criteria for assessing fusion [17,18]. Preoperative and postoperative lumbar spine images were matched to determine the sagittal alignment of the specific involved spinal level, with follow-up conducted for one year. Figures 1, 2 display the measurement of spinopelvic parameters in a spondylolisthesis patient pre- and post-TLIF.

Data analysis

Data were analyzed using the SPSS software version 2.0. Descriptive statistics were used to summarize patient demographics and baseline characteristics. Paired t-tests were used to compare preoperative and postoperative VAS, ODI scores, and radiological outcomes. A p value of less than 0.05 was considered statistically significant.

## Results

The mean age of the participants was 36.9 ± 12.2 years. The study included 12 (26.7%) males and 33 (73.3%) females. The average operating time is two hours with 200-300 mL of blood loss. Most patients presented with low back pain with radiculopathy. Table 1 represents the variations in PI after TLIF surgery. The PI from baseline 60.35 ± 3.11 changed to 59.35 ± 3.11, 60.35 ± 3.11, and 59.4 ± 3.08 at three-month, six-month, and one-year follow-ups. There was no significant change from the preoperative measurement to the last follow-up (p > 0.05).

**Table 1:** Evaluating the variation in PI from the baseline measurement to each follow-up assessment PI: pelvic incidence

Frequency	PI
Preoperative	60.35 ± 3.11
Three-month postop assessment	59.35 ± 3.11
Six-month postop assessment	60.35 ± 3.11
One-year postop assessment	59.4 ± 3.08

Table 2 depicts variations in SL, LL, and SVA. The average preoperative SL was -8.05, LL was 53.25, and SVA was -1.0. At the third-month follow-up, SL was 10.64, LL was 53.83, and SVA was 1.1, respectively (p < 0.05). At the last follow-up, SL was 15.51, LL was 52.16, and SVA was 2.5, respectively (p < 0.05).

**Table 2:** Evaluating the variations in SL, LL, and SVA from the baseline measurement to each follow-up assessment SL: segmental lordosis; LL: lumbar lordosis; SVA: sagittal vertical axis

Follow-up	SL	LL	SVA
Preoperative	-8.05 ± 1.78	-53.25 ± 7.4	-1.0 ± 0.5
Three months	-10.64 ± 1.68	-53.83 ± 8.52	1.1 ± 0.5
Last follow-up	-15.51 ± 1.79	-52.16 ± 6.96	2.5 ± 0.3

The preoperative VAS and ODI significantly decreased from baseline 6.4 ± 1.2 to 4 ± 0.8, 1.8 ± 0.8, and 0.4 ± 0.5 at three-month, six-month, and one-year follow-ups. The ODI score changed from 32.6 ± 4.3 (baseline) to 9.08 ± 2.6, 7.09 ± 3.2, and 5.23 ± 2.6 at three-month, six-month, and one-year follow-ups, respectively. Since the p value is less than 0.05, VAS and ODI scores were statistically improved after the surgery (Table 3).

**Table 3:** Comparison of preoperative and postoperative VAS and ODI scores VAS: Visual Analog Scale; ODI: Oswestry Disability Index

Follow-up	VAS	ODI
Preoperative	6.4 ± 1.2	32.6 ± 4.3
Three months	4 ± 0.8	9.08 ± 2.6
Six months	1.8 ± 0.8	7.09 ± 3.2
One year	0.4 ± 0.5	5.23 ± 2.6

Table 4 evaluates clinical outcomes using Macnab criteria at three-month, six-month, and one-year follow-ups. At the three-month postop appointment, six (13.3%) had poor outcomes, seven patients (15.6) had fair outcomes, and 32 patients (61.1%) had good-to-excellent outcomes after TLIF surgery. At the six-month follow-up, four patients (8.9%) had poor outcomes, seven patients (15.6%) had fair outcomes, and 34 patients (75.5%) had good-to-excellent outcomes. At the final follow-up, seven patients (15.6%) had fair outcomes, and 38 patients (84.4) had good-to-excellent outcomes after TLIF surgery.

**Table 4:** Clinical outcomes (Macnab criteria) at all follow-ups

Clinical outcome	Three months	Six months	One year
Poor	6 (13.3)	4 (8.9)	-
Fair	7 (15.6)	7 (15.6)	7 (15.6)
Good	27 (60)	28 (62.2)	20 (44.4)
Excellent	5 (11.1)	6 (13.3)	18 (40)

Table 5 represents radiological fusion using modified Lee criteria. At the final follow-up, 44.4% of the patients had a definitive fusion, and 18 patients (40%) had probable fusion. Only seven patients (15.5%) had pseudoarthrosis.

**Table 5:** Evaluation of fusion using modified Lee criteria

Fusion status	Number of patients	Percentage (%)
Definitive fusion	20	44.44%
Probable fusion	18	40%
Possible pseudoarthrosis	5	11.11%
Definitive pseudoarthrosis	2	4.44%

Table 6 represents the correlation between ODI and PI/SVA, as the p value is >0.05. There is no correlation between the clinical and radiological outcomes after TLIF surgery.

**Table 6:** correlation between PI vs. ODI and SVA vs. ODI PI: pelvic incidence; ODI: Oswestry Disability Index; SVA: sagittal vertical axis

Follow-up		PI vs. ODI	SVA vs. ODI
Baseline	R-value	0.0503	0.0188
	P-value	0.724	0.902
Three months	R-value	0.133	-0.1606
	P-value	0.383	0.293
Six months	R-value	0.133	0.118
	P-value	0.383	0.440
One year	R-value	0.118	0.131
	P-value	0.44	0.391

## Discussion

The patient demographics showed a mean age of 36.9 ± 12.2 years, with a majority being female (73.3%). This gender distribution is consistent with the general prevalence of spine-related issues, which tend to affect females more frequently [11,19]. The Meyerding grading system classified the degree of spondylolisthesis, with most participants categorized as grade I (37.8%). This distribution corresponds with findings from other studies, such as those by Ould-Slimane et al. and Kakadiya et al., indicating that the severity of spondylolisthesis in our cohort is representative of broader patient populations [11,19].

L4-L5 levels were the most commonly affected in this study 21 (46.7%). The least amount, about 15.6%, belongs to L3-L4, and about 37.8% of the study participants belong to L5-S1. In the current study, most patients (77.8%) presented with low lumbar pain with radiculopathy. These presenting symptoms are similar to those reported in other studies, such as those by Ould-Slimane et al. and Kakadiya et al., reinforcing the typical clinical presentation of spondylolisthesis [11,19]. The operating times were well-distributed, averaging two hours, with around 200-300 mL blood loss.

Radiological parameters, including PT, SS, SL, LL, and SVA, showed significant postoperative improvements. There was not much change in PI postoperatively, as it is assumed to be a fixed value [20]. The observed PT, SS, SL, and LL improvements suggest that TLIF effectively corrects spinopelvic balance, enhancing spinal stability.

Clinical outcomes also demonstrated significant improvements, with reduced leg and back pain measured by VAS and ODI scores. The study reported significant decreases in pain and disability scores post-TLIF [16]. The improvements in VAS and ODI scores confirm the procedure's effectiveness in alleviating patient symptoms and enhancing quality of life.

The Macnab criteria further evaluated clinical outcomes, revealing progressive improvements over time. At one-year follow-up, 84.4% reported either excellent or good outcomes based on Macnab criteria, and 84.4% showed definitive fusion based on modified Lee criteria [17,18]. These results suggest that functional improvement continues to develop over time, and TLIF is associated with substantial positive outcomes.

The improvements observed in radiological parameters, such as PT, SS, SL, and SVA, highlight the positive impact of TLIF on spinal alignment. Clinically, significant reductions in pain (VAS scores) and disability (ODI scores) underscore the procedure's effectiveness in relieving symptoms and enhancing quality of life. These findings are particularly relevant for practitioners considering surgical options for patients with spondylolisthesis, as TLIF demonstrates robust benefits across multiple measures of patient health [21,22].

Despite the promising outcomes, several limitations must be acknowledged. The relatively small sample size and the observational study design may limit the generalizability and causal inference of the findings. Additionally, potential selection bias during participant recruitment and variations in imaging techniques could introduce biases and measurement inconsistencies. Addressing these limitations in future research through larger sample sizes, randomized controlled trials, and standardized methodologies would strengthen the validity and reliability of the findings.

## Conclusions

This study provides valuable insights into the effectiveness of TLIF surgery in addressing spondylolisthesis, both in terms of radiological and functional outcomes. However, there was no correlation between improvement in functional and radiological parameters (PI vs. Macnab criteria, SVA vs. Macnab criteria). TLIF appears to offer significant improvements in patient well-being and quality of life. These findings contribute to the understanding of TLIF's suitability as a treatment for spondylolisthesis and can inform clinical practice, ultimately benefiting patients suffering from this condition.
